# Adipose tissue insulin resistance index was inversely associated with gluteofemoral fat and skeletal muscle mass in Japanese women

**DOI:** 10.1038/s41598-024-67184-6

**Published:** 2024-07-16

**Authors:** Satomi Minato-Inokawa, Mari Honda, Ayaka Tsuboi-Kaji, Mika Takeuchi, Kaori Kitaoka, Miki Kurata, Bin Wu, Tsutomu Kazumi, Keisuke Fukuo

**Affiliations:** 1 Research Institute for Nutrition Sciences, Mukogawa Women’s University, 6-46, Ikebiraki-cho, Nishinomiya, Hyogo 663-8558 Japan; 2 Laboratory of Community Health and Nutrition, Department of Bioscience, Graduate School of Agriculture, Ehime University, Matsuyama, Ehime Japan; 3 Open Research Center for Studying of Lifestyle-Related Diseases, Mukogawa Women’s University, Nishinomiya, Hyogo Japan; 4 Department of Health, Sports, and Nutrition, Faculty of Health and Welfare, Kobe Women’s University, Kobe, Hyogo Japan; 5 Department of Nutrition, Osaka City Juso Hospital, Osaka, Japan; 6 Department of Advanced Epidemiology, Noncommunicable Disease (NCD) Epidemiology Research Center, Shiga University of Medical Science, Otsu, Shiga Japan; 7 Department of Food Sciences and Nutrition, Mukogawa Women’s University, Nishinomiya, Hyogo Japan; 8 Department of Endocrinology, First Affiliated Hospital of Kunming Medical University, Kunming, Yunnan China; 9 Department of Medicine, Kohan Kakogawa Hospital, Kakogawa, Hyogo Japan

**Keywords:** Adipose tissue insulin resistance, Homeostasis-model assessment-insulin resistance, Body composition, Leg fat, Trunk fat, Skeletal muscle, Medical research, Risk factors

## Abstract

Associations of adipose tissue insulin resistance index (AT-IR, a product of fasting insulin and free fatty acids) with body fat mass and distribution and appendicular skeletal muscle mass (ASM) were compared with results of homeostasis-model assessment-insulin resistance (HOMA-IR) in 284 Japanese female university students and 148 their biological mothers whose BMI averaged < 23 kg/m^2^. Although mothers compared with daughters had higher BMI, body fat percentage, trunk fat to body fat (TF/BF) ratio and lower leg fat to body fat (LF/BF), AT-IR and HOMA-IR did not differ. We had multivariable linear regression analyses which included TF/BF ratio, LF/BF ratio, weight-adjusted ASM (%ASM), height-adjusted ASM index (ASMI), fat mass index (FMI), and body fat percentage. In young women, AT-IR was independently associated with LF/BF ratio (Standardized β [Sβ]: − 0.139, *p* = 0.019) and ASMI (Sβ: − 0.167, *p* = 0.005). In middle-aged women, LF/BF ratio (Sβ: − 0.177, *p* = 0.049) and %ASM (Sβ: − 0.205, *p* = 0.02) emerged as independent determinants of AT-IR. HOMA-IR was associated with TF/BF ratio and FMI, a proxy of abdominal and general adiposity, respectively, in both young and middle-aged women. The inverse association of AT-IR with leg fat may support the notion that limited peripheral adipose storage capacity and small skeletal muscle size are important etiological components in insulin-resistant cardiometabolic disease in Japanese women.

## Introduction

Insulin resistance and impaired insulin secretion are major hallmarks of type 2 diabetes. Fat accumulation in the upper body (abdominal/truncal obesity) is associated with insulin resistance, type 2 diabetes and cardiovascular disease^1–5^. In contrast, fat accumulation in the lower body (gluteofemoral/leg region) shows favorable associations with cardiometabolic health when adjusted for overall fat mass^1–5^.

Lipodystrophies are relatively rare and characterized by impairments in white adipose tissue function and its mass or distribution^6^. Genome-wide association studies focusing on insulin resistance^7,8^ revealed that elevated insulin resistance scores were associated with lower BMI and lower leg subcutaneous fat mass, a subtle lipodystrophy-like phenotype, and suggest that the link may be associated with the impaired capacity to adequately expand the peripheral adipose tissue compartments. Han et al.^9^ reported that low leg fat to total body fat ratio is the strongest independent predictor of cardiometabolic health in normal-weight subjects whereas it is not a significant determinant of metabolic risk in obese subjects. We showed that lower birthweight^10^ and a positive family history of type 2 diabetes^11^ were associated with low leg fat mass in young Japanese women.

Skeletal muscle is the primary tissue responsible for insulin-dependent glucose uptake in vivo^12^. Studies demonstrated that low skeletal muscle mass was associated with insulin resistance, metabolic syndrome, and type 2 diabetes in adolescents or youth, general populations, and older adults^13–18^.

Although the gold standard to measure multi-tissue insulin resistance is a euglycemic-hyperinsulinemic clamp procedure and stable isotopically labeled tracer infusions, there are two simple and reliable indices of insulin resistance: homeostasis model assessment- insulin resistance (HOMA-IR)^19^ and adipose tissue- insulin resistance (AT-IR)^20^. We showed that AT-IR (a product of fasting insulin and fasting free fatty acid [FFA]) may be useful in assessing adipose insulin resistance even in women without diabetes and obesity^21^. AT-IR was associated positively with trunk/leg fat ratio, fasting glucose, and triglyceride in young and middle-aged women. In middle-aged women, AT-IR was also associated with waist circumference and blood pressure^21^. As HOMA-IR (a product of fasting insulin and fasting glucose) is an index that focuses on glucose metabolism which encompasses hepatic and muscle tissues, we tested whether the associations with body composition (skeletal muscle mass, body fat mass, and distribution) may be different between AT-IR and HOMA-IR in young and middle-aged Japanese women, in whom confounding factors were so scarce^22^.

## Methods

We cross-sectionally studied 284 Japanese female students (74 collegiate athletes and 210 non-athletes) and 148 their biological mothers, whose details have been reported elsewhere^21–24^. Athletes were students of the Department of Health and Sports Sciences and nonathletes were students of the Department of Food Sciences and Nutrition. Although our previous study on AT-IR^21^ excluded athletic students to avoid the effects of endurance training on AT-IR^24^, the present study included athletic students to obtain a wider range of AT-IR and skeletal muscle mass^24^. Women with clinically diagnosed acute or chronic diseases, those on hormonal contraception, and those on a diet to lose weight were excluded from the study. Nobody reported receiving any medications or having regular supplements. The study was in accordance with the Helsinki Declaration. All subjects were recruited as volunteers and gave written consent after the experimental procedure had been explained.

After a 12-h overnight fast, participants underwent blood sampling and measurements of height, weight, and body composition by whole-body dual-energy X-ray absorptiometry (DXA) as described later. Glucose was determined by the hexokinase/glucose-6-phosphate dehydrogenase method (inter-assay coefficient of variation (CV) < 2%). Serum insulin was measured by an ELISA method with a narrow specificity excluding des-31, des-32, and intact proinsulin (interassay CV < 6%). Serum triglyceride concentrations were measured using an autoanalyzer (AU5232; Olympus, Tokyo, Japan). FFA was measured using enzymatic colorimetric methods (Wako, Tokyo, Japan). HOMA-IR was calculated as fasting insulin (µU/mL) × glucose (mg/dL)/405^19^ and AT-IR as a product of fasting insulin (µU/mL) and fasting FFA (mEq/L)^20^.

Fat mass, lean mass and total mass for arms, legs and the trunk all in kilograms were measured using whole-body DXA (Hologic QDR-2000, software version 7.20D, Waltham, MA) as previously reported^2,22,23^. The leg region included the entire hip, thigh, and legs. General adiposity was assessed using height-adjusted and weight-adjusted body fat; fat mass index (FMI) and body fat (BF) percentage (%), respectively. As elevated trunk/leg fat ratio results from elevated trunk fat and/or reduced leg fat, fat mass in the trunk and legs were adjusted by total body fat as follows: leg fat (LF) to BF ratio was calculated as LF divided by BF × 100 and trunk fat (TF) to BF ratio as TF divided by BF × 100. Because lean mass in arms and legs represents skeletal muscle mass, a sum of the two was used as appendicular skeletal muscle mass (ASM). ASM index (ASMI) was calculated as ASM in kilograms divided by squared height in meters. ASM% was calculated as ASM (kg) divided by body weight (kg) × 100.

Data were presented as mean ± SD. Correlations of AT-IR and HOMA-IR with body composition measures were evaluated by Pearson’s correlation analysis. To evaluate the most important determinants of AT-IR and HOMA-IR, a stepwise multivariate linear regression analyses were performed. Independent variables included were FMI, body fat%, LF/BF ratio, TF/BF ratio, ASMI and ASM%. Differences between the two groups were analyzed with a t-test. Differences among three groups were analyzed by analysis of variance and then Bonferroni's multiple comparison procedure. A two-tailed value of *p* < 0.05 was considered significant. Statistics were performed with SPSS system 23.0 (SPSS Inc, Chicago, IL).

### Ethics approval

The study was approved by the Ethics Committees of the Mukogawa Women’s University (No. 07-28 on 19/02/2008).

## Results

Middle-aged compared with young women had higher BMI, FMI, and %body fat and lower ASMI and ASM% (Table 1). They also had higher fasting glucose, triglyceride, absolute fat mass in arms, the trunk, and total body whereas absolute fat in legs, fasting insulin, AT-IR, and HOMA-IR did not differ between two groups of women. In middle-aged women, LF/BF ratio was lower, and TF/BF ratio, %arm fat, %leg fat, and %trunk fat were higher. The trunk/leg fat ratio and %trunk fat to %leg fat ratio were also higher in middle-aged women.

**Table 1:** Body composition and two indices of insulin resistance studied in young and middle-aged Japanese women.

	Young	Middle-aged	*p* values
	n = 248	n = 148	
Age (years)	20.7 ± 1.2	49.8 ± 3.6	< 0.001
Height (cm)	161.0 ± 6.3	156.7 ± 5.2	< 0.001
Weight (kg)	53.9 ± 7.4	53.9 ± 7.0	0.984
Body mass index (kg/m^2^)	20.7 ± 2.2	22.0 ± 2.8	< 0.001
Arm fat (kg)	1.1 ± 0.6	1.5 ± 0.7	< 0.001
Leg fat (kg)	5.5 ± 1.6	5.4 ± 1.7	0.465
Trunk fat (kg)	6.7 ± 2.3	8.8 ± 3.4	< 0.001
Body fat (kg)	13.9 ± 4.3	16.3 ± 5.7	< 0.001
Trunk/leg fat ratio	1.23 ± 0.24	1.64 ± 0.39	< 0.001
Leg fat/body fat (%)	40.0 ± 4.4	33.6 ± 5.0	< 0.001
Trunk fat/body fat (%)	48.1 ± 4.0	53.3 ± 5.0	< 0.001
%Arm fat (%)	22.2 ± 8.4	27.8 ± 8.8	< 0.001
%Leg fat (%)	28.7 ± 5.7	30.3 ± 6.7	0.010
%Trunk fat (%)	26.7 ± 6.8	32.9 ± 8.7	< 0.001
%Body fat	25.9 ± 5.9	30.1 ± 7.3	< 0.001
%Trunk/%leg fat ratio	0.93 ± 0.14	1.08 ± 0.18	< 0.001
Fat mass index (kg/m^2^)	5.4 ± 1.7	6.67 ± 2.37	< 0.001
ASMI (kg/m^2^)	6.18 ± 0.75	5.99 ± 0.53	0.002
%ASM (%)	29.9 ± 2.9	27.5 ± 2.9	< 0.001
Fasting glucose (mg/dL)	83 ± 7	89 ± 14	< 0.001
Fasting insulin (µU/mL)	5.9 ± 3.2	5.4 ± 2.8	0.143
Fasting triglyceride (mg/dL)	58 ± 34	81 ± 36	< 0.001
AT-IR	3.1 ± 2.5	3.2 ± 2.1	0.578
HOMA-IR	1.2 ± 0.7	1.2 ± 0.7	0.985

Mean ± SD. ASM, appendicular skeletal muscle mass; %ASM, percentage ASM; AT-IR, adipose tissue-insulin resistance; HOMA-IR, homeostasis model assessment-insulin resistance.

In young Japanese women (Table 2), HOMA-IR was associated with 11 of 12 adiposity and body composition measures studied, except for ASMI, which showed no association with HOMA-IR. In contrast, AT-IR was not associated with BMI, FMI, and % body fat and the association with TF/BF ratio did not reach statistical significance (*p* = 0.08). AT-IR was associated inversely with only three variables, i.e., LF/BF ratio, ASMI, and %ASM.

**Table 2:** Correlation coefficients (r) of adipose tissue-insulin resistance (AT-IR) and homeostasis model assessment-insulin resistance (HOMA-IR) in young Japanese women.

	AT-IR		HOMA-IR	
	r	*p* values	r	*p* values
Body mass index	− 0.097	0.104	**0**.**191**	< **0**.**001**
Arm fat	0.023	0.705	**0**.**202**	< **0**.**001**
Leg fat	− 0.100	0.097	**0**.**145**	< **0**.**001**
Trunk fat	− 0.003	0.962	**0**.**231**	< **0**.**001**
Body fat	− 0.034	0.566	**0**.**210**	< **0**.**001**
Leg fat/body fat	− **0**.**159**	**0**.**008**	− **0**.**174**	< **0**.**001**
Trunk fat/body fat	0.104	0.083	**0**.**150**	**0**.**001**
%Arm fat	0.092	0.124	**0**.**170**	< **0**.**001**
%Leg fat	0.034	0.569	**0**.**130**	**0**.**004**
%Trunk fat	0.078	0.195	**0**.**191**	< **0**.**001**
%Body fat	0.065	0.277	**0**.**180**	< **0**.**001**
Fat mass index	0.012	0.847	**0**.**211**	< **0**.**001**
ASMI	− **0**.**183**	**0**.**002**	0.033	0.471
%ASM	− **0**.**122**	**0**.**041**	− **0**.**139**	**0**.**002**
AT-IR	1.000		**0**.**475**	< **0**.**001**

Abbreviations are the same as in Table 1. Bold numbers denote significant associations.

In middle-aged women (Table 3), both AT-IR and HOMA-IR showed significant associations with all measures of body composition studied, except for ASMI, which showed no association in two groups of women. Associations between AT-IR and HOMA-IR were stronger in middle-aged compared with young women (r: 0.739 vs. 0.475, *p* = 0.003).

**Table 3:** Correlation coefficients (r) of adipose tissue-insulin resistance (AT-IR) and homeostasis model assessment-insulin resistance (HOMA-IR) in middle-aged Japanese women.

	AT-IR		HOMA-IR	
	r	*p* values	r	*p* values
Body mass index	0.206	0.012	0.431	< 0.001
Arm fat	0.202	< 0.001	0.368	< 0.001
Leg fat	0.155	0.064	0.311	< 0.001
Trunk fat	0.291	< 0.001	0.472	< 0.001
Body fat	0.256	0.002	0.437	< 0.001
Leg fat/body fat	− 0.271	0.001	− 0.360	< 0.001
Trunk fat/body fat	0.275	0.001	0.367	< 0.001
%Arm fat	0.233	0.005	0.347	< 0.001
%Leg fat	0.194	0.020	0.311	< 0.001
%Trunk fat	0.299	< 0.001	0.440	< 0.001
%Body fat	0.279	0.001	0.419	< 0.001
Fat mass index	0.264	0.001	0.447	< 0.001
ASMI	− **0**.**045**	**0**.**591**	**0**.**114**	**0**.**174**
%ASM	− 0.286	0.001	− 0.412	< 0.001
AT-IR	1.000		0.739	< 0.001

Abbreviations are the same as in Table 1. Bold numbers denote nonsignificant association.

We had multivariate linear regression analysis for AT-IR and HOMA-IR (Table 4). All models included the same six independent variables: FMI, body fat%, LF/BF ratio, TF/BF ratio, %ASM and ASMI. AT-IR was independently associated with LF/BF ratio and ASMI in young women and LF/BF ratio and %ASM in middle-aged women. For HOMA-IR, TF/BF ratio and FMI emerged as independent determinants in both young and middle-aged women.

**Table 4:** Multivariate linear regression analysis for adipose tissue insulin resistance index (AT-IR) and homeostasis model assessment-insulin resistance (HOMA-IR) in young and middle-aged Japanese women.

AT-IR	Young		Middle-aged	
	Standardized β	*p* values	Standardized β	*p* values
Leg fat/body fat	− 0.139	0.019	− 0.177	0.049
ASMI	− 0.167	0.005		
%ASM			− 0.205	0.023
Cumulative R^2^		0.046		0.094
HOMA-IR				
Trunk fat/body fat	0.148	0.018	0.214	0.010
Fat mass index	0.128	0.040	0.355	< 0.001
Cumulative R^2^		0.044		0.226

All models included the same six independent variables: fat mass index, body fat percentage, leg fat/body fat ratio, trunk fat/body fat ratio, %ASM and ASMI. Abbreviations are the same as in Table 1.

**Figure 1:**
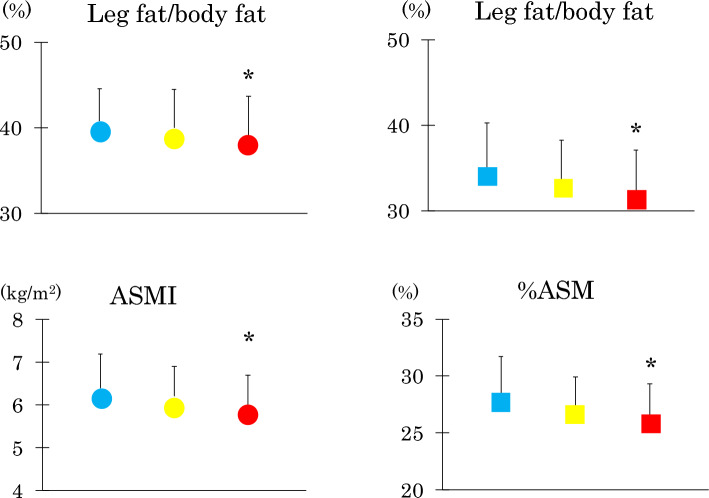
Leg fat/body fat ratio, appendicular skeletal muscle mass (ASM) index (ASMI) in young women (circles) and percentage (%ASM) in middle-aged women (squares). They were divided according to respective tertile of adipose tissue-insulin resistance index (AT-IR). Blue, yellow and red symbols represent the low, median and high tertile, respectively. Mean ± SD. *p < 0.05 or less versus the low tertile by Bonferroni’s multiple comparison procedure.

Young and middle-aged women were divided into three groups according to respective AT-IR tertile (Fig. 1). As AT-IR increased, there were stepwise decreases in LF/BF ratio in two groups of women, ASMI in young women and %ASM in middle-aged women. As athletic compared with non-athletic students had higher muscle mass and lower AT-IR^24^, the percentage of athletic students decreased as AT-IR increased (40.4, 25.5 and 12.5% in the low, medium, and high tertile, respectively, *p* < 0.001). Therefore, we analyzed non-athletic students alone. In multivariate linear regression analysis which included the same six independent variables as in Table 4, the LF/BF ratio (standardized β: − 0.142, *p* = 0.01) emerged as a single independent determinant of AT-IR (R^2^ = 0.015).

Next, to see associations with fasting glucose, insulin, and TG, the two groups of women were divided into three groups according to the respective tertile of LF/BF and TF/BF ratio (Table 5). In young women, the LF/BF ratio was associated with two variables (fasting insulin and TG) whereas the TF/BF ratio was associated with fasting TG alone. In middle-aged women, the LF/BF ratio was associated with all three variables whereas the TF/BF ratio was associated with two variables (fasting insulin and TG).

**Table 5:** Associations of leg fat (relative to body fat) and trunk fat (relative to body fat) with fasting glucose, insulin, and triglyceride in young and middle-aged Japanese women.

LF/BF tertile: young	Low	Median	High	#
Fasting glucose (mg/dL)	82 ± 6	82 ± 6	84 ± 8	
Fasting insulin (µU/mL)	6.6 ± 3.6	5.7 ± 2.9	5.4 ± 3.0	b
Fasting triglyceride (mg/dL)	64 ± 29	55 ± 24	55 ± 45	
log triglyceride	1.77 ± 0.18	1.71 ± 0.17	1.69 ± 0.18	a,b

LF/BF tertile: middle-aged	Low	Median	High	
Fasting glucose (mg/dL)	93 ± 19	89 ± 11	86 ± 8	b
Fasting insulin (µU/mL)	6.4 ± 3.1	5.7 ± 2.5	4.1 ± 2.0	b,c
Fasting triglyceride (mg/dL)	95 ± 37	85 ± 36	66 ± 29	b,c

TF/BF tertile: young	Low	Median	High	
Fasting glucose (mg/dL)	83 ± 7	83 ± 7	82 ± 6	
Fasting insulin (µU/mL)	5.7 ± 3.0	5.4 ± 2.8	6.5 ± 3.7	
Fasting triglyceride (mg/dL)	52 ± 18	59 ± 46	64 ± 31	b

TF/BF tertile: middle-aged	Low	Median	High	
Fasting glucose (mg/dL)	88 ± 20	87 ± 9	92 ± 10	
Fasting insulin (µU/mL)	4.3 ± 1.9	5.6 ± 2.8	6.3 ± 3.1	a,b
Fasting triglyceride (mg/dL)	68 ± 32	88 ± 40	89 ± 32	a,b

Mean ± SD. #: a: low versus median, b: low versus high, c: median versus high at *p* < 0.05 or less by Bonferroni’s multiple comparison procedure. LF/BF: leg fat/body fat, TF/BF: trunk fat/body fat.

## Discussion

The present study has shown that in young women, adipose tissue insulin resistance (measured by AT-IR) was not associated with body fat mass (measured by FMI and body fat%) but with body fat distribution although it was associated with fat mass and distribution in middle-aged women. Adipose insulin resistance was independently associated not with trunk fat but with leg (gluteofemoral subcutaneous) fat (inverse) in young Japanese women. Further, adipose insulin resistance was associated with skeletal muscle (inverse) in young women. Finally, inverse associations of AT-IR with gluteofemoral fat and skeletal muscle mass were confirmed in middle-aged Japanese women, whose BMI averaged 22 kg/m^2^ and HOMA-IR 1.2.

There are a limited number of studies that employed DXA to measure leg fat and evaluated body fat distribution by LF/BF ratio. For example, studies including ours^9,23^ reported that LF/BF ratio is a strong independent predictor of cardiometabolic health in normal-weight subjects. Zhang et al.^25^ studied participants in the US National Health and Nutrition Examination Survey and found that LF/BF ratio was inversely associated with the risk of metabolic syndrome. Kim and Lee^26^ studied Korean people and found that a lower LF/TF ratio was markedly associated with a higher risk of non-alcoholic fatty liver disease, a member of metabolic syndrome.

Obesity, especially upper body (truncal or abdominal), is associated with insulin resistance in the liver, muscle, and adipose tissue^27^. HOMA-IR (a proxy of liver and muscle insulin resistance) consistently showed a positive association with measures of general and truncal adiposity (measured by FMI and TF/BF ratio, respectively) in the present study. However, elevated adipose insulin resistance was associated with lower leg (gluteofemoral) fat, a subtle lipodystrophy-like phenotype, in young and middle-aged Japanese women without obesity and diabetes in the present study. These observations may be consistent with the results of genome-wide association studies focusing on insulin resistance^7,8^ that elevated insulin resistance scores were associated with lower leg subcutaneous fat mass. The inverse association of AT-IR with leg fat in Japanese women may support the notion that the limited storage capacity of peripheral adipose tissue is an important etiological component in insulin-resistant cardiometabolic disease.

A systematic review and meta-analysis^28^ suggested that insulin may be facilitative in human skeletal muscle anabolism and that the effects of insulin in reducing muscle protein breakdown are blunted in older people and those with insulin resistance. Consistent with these notions, an association of HOMA-IR with low relative muscle mass was reported in some studies^13–16,29^ and confirmed in the present study (assessed by %ASM). As far as we know, the present study is the first to demonstrate an inverse association of adipose tissue insulin resistance with skeletal muscle mass even in young lean Japanese women. It is well known that an increased flux of fatty acids from the adipose tissue may contribute to increased fat storage in the liver and in skeletal muscle and impaired insulin signaling in skeletal muscle^30^. Therefore, our findings may be associated with the study by Stephens et al.^31^ who suggested that excess lipid availability induced insulin resistance of skeletal muscle glucose metabolism as well as anabolic resistance of ingested amino acid metabolism in healthy young men.

Zhu et al.^32^ examined racial/ethnic disparities in the prevalence of diabetes by BMI category in a large cohort of 4.9 million adults. They found that Asians had a higher burden of diabetes at lower BMIs than whites. They suggested a higher body fat percentage and visceral fat at a given BMI and poor insulin secretion as the underlying mechanisms^32^. The present study suggests that a subtle lipodystrophy-like phenotype associated with limited storage capacity of leg adipose tissue may be one of underlying mechanisms contributing to the natural history of diabetes incidence in Japanese.

We reported in another set of daughter-mother pairs that the mean BMI of mothers increased from 20.0 kg/m^2^ at 18 years old to 21.8 kg/m^2^ at 48 years old^33^. The BMI of 18-year-old daughters (20.2 kg/m^2^) was associated not only with their mothers’ BMI when they were 18 years old but with the current BMI of their mothers. In middle-aged compared with young Japanese women in the present study, higher body fat resulted from higher trunk fat with similar leg fat. These findings suggest that increases in body fat from young to middle-aged Japanese women resulted mainly from increases in trunk (abdominal) fat, a marker of ectopic fat accumulation^34^, suggesting reduced leg fat expandability in Japanese women.

The trunk/leg (limb) fat ratio and %trunk fat/%leg fat ratio were higher in middle-aged than young women. These DXA-derived ratios may be valuable tools for identifying individuals with lipodystrophy-like phenotypes^35,36^. The prevalence of the %trunk fat/%leg fat ratio > 1.2, a cutoff value of the high ratio for women^36^, was approximately 13% of participants, in whom women had a mean BMI of 26.2 kg/m^2^, in the UK Biobank^36^. In the present study, it was much higher (26%) in middle-aged Japanese lean women, whose BMI averaged 22.0 kg/m^2^.

Elevated trunk/leg (limb) fat ratio was repeatedly confirmed in adolescents, middle-aged, and elderly Asians compared with White women^37–39^. However, the elevated trunk/leg fat ratio was not due to elevated trunk fat but reduced peripheral fat. In a prior study comparing elderly Japanese American women to White women with comparable BMI, total body, and trunk fat (14.3 and 14.9 kg, *p* = 0.68), an elevated trunk/leg fat ratio was due to reduced leg fat (7.7 and 10.4 kg, *p* = 0.0007)^39^. These observations may indicate reduced leg fat expandability, and subtle lipodystrophy-like phenotypes, in Asian women and those of Asian ancestry.

The strength of the present study includes a homogeneous study population with few confounding factors and accurate and reliable measures of body composition by DXA. Several limitations of this study warrant consideration. The cross-sectional design complicates the drawing of causal inferences, and a single measurement of biochemical variables may be susceptible to short-term variation, which would bias the results toward the null. We used crude measures of IR, which may be less accurate. Statistical power was not calculated. As we studied young and middle-aged Japanese women, results may not be generalized to other genders, age populations, races or ethnicities.

In conclusion, inverse associations of adipose tissue insulin resistance with subcutaneous leg fat and skeletal muscle mass may support the notion that the limited storage capacity of peripheral adipose tissue and small skeletal muscle size may be important etiological components in insulin-resistant cardiometabolic disease in Japanese women. However, an association with skeletal muscle warranted further studies.

## Data Availability

The datasets used and/or analyzed during the current study are available from the corresponding author on reasonable request.
